# Coal Fly Ash Ceramics: Preparation, Characterization, and Use in the Hydrolysis of Sucrose

**DOI:** 10.1155/2014/154651

**Published:** 2014-07-03

**Authors:** Ricardo Pires dos Santos, Jorge Martins, Carlos Gadelha, Benildo Cavada, Alessandro Victor Albertini, Francisco Arruda, Mayron Vasconcelos, Edson Teixeira, Francisca Alves, José Lima Filho, Valder Freire

**Affiliations:** ^1^Computer Engineering, Federal University of Ceará, 62010-560 Sobral, CE, Brazil; ^2^Institute of Chemistry and Geosciences, Federal University of Pelotas, 96160-000 Pelotas, RS, Brazil; ^3^Department of Molecular Biology, Federal University of Paraíba, 58059-900 João Pessoa, PB, Brazil; ^4^Laboratory of Biologically Active Molecules, Federal University of Ceará, 60440-970 Fortaleza, CE, Brazil; ^5^Laboratory of Theoretical, Computational and Experimental Biophysics, Federal Rural University of Pernambuco, 52171-900 Recife, PE, Brazil; ^6^Integrated Laboratory of Biomolecules, Federal University of Ceará, 60430-160 Fortaleza, CE, Brazil; ^7^Laboratory of Immunopathology Keizo Asami (LIKA), Federal University of Pernambuco, 50780-901 Recife, PE, Brazil; ^8^Department of Physics, Federal University of Ceará, 60455-900 Fortaleza, CE, Brazil

## Abstract

Coal ash is a byproduct of mineral coal combustion in thermal power plants. This residue is responsible for many environmental problems because it pollutes soil, water, and air. Thus, it is important to find ways to reuse it. In this study, coal fly ash, obtained from the Presidente Médici Thermal Power Plant, was utilized in the preparation of ceramic supports for the immobilization of the enzyme invertase and subsequent hydrolysis of sucrose. Coal fly ash supports were prepared at several compaction pressures (63.66–318.30 MPa) and sintered at 1200°C for 4 h. Mineralogical composition (by X-ray diffraction) and surface area were studied. The ceramic prepared with 318.30 MPa presented the highest surface area (35 m^2^/g) and amount of immobilized enzyme per g of support (76.6 mg/g). In assays involving sucrose inversion, it showed a high degree of hydrolysis (around 81%) even after nine reuses and 30 days' storage. Therefore, coal fly ash ceramics were demonstrated to be a promising biotechnological alternative as an immobilization support for the hydrolysis of sucrose.

## 1. Introduction

Coal ash is a byproduct of mineral coal combustion in thermal power plants [1, 2]. Its physical and chemical characteristics depend on the origin of the coal as well as its burning process in the power plant [3]. However, the main chemical elements usually found in coal ash are silicon, calcium, aluminum, iron, magnesium, sulfur, carbon, and several trace elements [4]. Based on the burning process, coal ash is divided into three types: slag, bottom ash, and fly ash [5]. Fly ash is a residue collected by electrostatic precipitators, mechanical filters, or it is simply ejected into the atmosphere [6]. It is composed mainly of hollow spherical particles known as cenospheres, which are particles of low density generally formed by glassy aluminum-silicate matrix, quartz, mullite, calcite, and magnetite [7]. Fly ash represents around 70% of the coal ash produced by thermal power plants [8]. The presence of this residue in the environment is highly detrimental as it pollutes soil, water, and air. In addition, it modifies the composition of the environmental medium through the insertion of potentially toxic substances [9]. However, based on its chemical composition, fly ash has been used as additive in the manufacture of cement and concrete, conventional ceramics, and vitroceramics, among other uses [10].

Enzymes, as mediators of synthesis and degradation in reactions, are more efficient catalysts than the conventional techniques used in chemical catalysis. Therefore, enzymes are largely exploited by biotechnology for the production of various substances [11]. When used in a soluble form, they retain some activity after the reaction; however, they cannot be recovered for later use. Thus, enzymes form a residual contaminant in the final product, and its removal involves extra steps of purification, thus elevating the costs of production [12]. A way to reduce this loss and increase productivity is by separating the enzyme from the product during the reaction and forming a polyphase system where one phase contains the enzyme (called support) and the other phase contains the product [13]. In this engineering design, the enzyme is immobilized on a support which becomes insoluble, albeit retaining its activity, enabling its reuse without contaminating the product [14]. A variety of insoluble materials can be used for immobilization, such as polymer matrices or normally inert inorganic materials [15].

Sucrose is a disaccharide found mainly in sugar cane and beet [16] and formed by molecules of glucose and fructose, with the elimination of water molecules [17]. Brazil is the world's largest producer of sucrose, with 420 million tons in 2005. It is also a major exporter, with 129 million tons in 2004 [18]. Sucrose can be hydrolyzed by dilution in acid or through the action of the enzyme invertase, which splits sucrose into glucose and fructose [19]. The products of partial or complete hydrolysis of sucrose are widely used in the food and pharmaceutical industries as a result of their high hygroscopicity and resistance to crystallization [18]. Enzymatic hydrolysis is the process that generates products with the lowest levels of hydroxymethylfurfural (HMF) and ash [20]. However, its use is limited by the high cost of invertase and its loss in the process of hydrolysis [18]. Thus, the hydrolysis of sucrose with invertase immobilized in bioreactors might positively address this limitation. Ceramics produced from the mixture of coal fly ash with other compounds have shown excellent results in the immobilization of invertase and subsequent hydrolysis of sucrose [21].

In this study, coal fly ash obtained from the Presidente Médici Thermal Power Plant was used without additives to prepare ceramic supports for the immobilization of invertase. The supports with the immobilized enzyme were used in experiments of sucrose hydrolysis. Physicochemical characterizations considered important to prepare the supports were also performed on the coal fly ash and ceramics.

## 2. Materials and Methods

### 2.1. Materials

Coal fly ash used to prepare the ceramics was collected by electrostatic precipitators of the B phase from the Presidente Médici Thermal Power Plant (UTPM) located in Candiota City, State of Rio Grande do Sul, Brazil. Approximately 2 kg of dried coal fly ash was collected in 2007 and filtered through a 2 mm sieve. Invertase (*β*-fructofuranosidase, E.C. 3.2.1.26) from baker's yeast (*S. cerevisiae* 200–300 units/mg solid), 3-aminopropyltriethoxysilane (APTES), 3,5-dinitrosalicylic acid (DNS), glutaraldehyde, and sucrose were acquired from Sigma-Aldrich Chemical Co. (St. Louis, MO, USA). Several analytical grade reagents were also used.

### 2.2. Preparation of Ceramics

Ceramics were prepared according to the methodology described by Albertini et al. [22]. Briefly, coal fly ash was uniaxially compacted in a steel mold by a SKAY 93 hydraulic press at 63.66 (C63), 127.32 (C127), 190.98 (C190), 254.64 (C254), and 318.30 (C318) MPa at 63.66 MPa steps for 10 s into pellets (diameter = 10 mm). Pressures above 318.30 MPa were not used because the steel mold had low tolerance for high levels of mechanical stress. Approximately 26.6 mg of fly ash was used for each pellet. Then, the pellets (green bodies) were sintered on air atmosphere at 1200°C for 4 h in an Elektro Therm Linn 1200 muffle-type oven. The muffle was programmed with a heating rate of 50°C min-1 to reach the sintering temperature. After cooling at room temperature, sintered pellets (ceramics) were washed with distilled water and dried in an Icamo stove at 60°C for 48 h. Ceramic samples were prepared without addition of binder in order to prevent the effect of thermal decomposition in the physical properties of the ceramic during the sintering step.

### 2.3. Characterization of Coal Fly Ash and Ceramics

#### 2.3.1. Mineralogical Analyses

The X-ray diffraction (XRD) patterns for the mineralogical analyses of coal fly ash and ceramics were obtained at room temperature (26.8°C) using a Shimadzu XRD-6000 powder diffractometer. Bragg-Bretano geometry was used with Cu-ka radiation. The tube was operated at 40 kV and 40 mA. The diffraction data were collected over the range of 10° ≤ 2*θ* ≤ 60° with 0.02° steps and an integration time of 2 s per point. Crystallinity (%) was obtained from the percentile ratio between the crystalline area and total area on the X-ray diffractogram [23]. X'Pert HighScore software (PANalytical) was used to identify crystalline phases; Rietica 1.7.7 software (Lucas Heights Research Laboratories) was used for structure refinement (Rietveld's method) and subsequent quantitative analysis.

#### 2.3.2. Thermogravimetric Analysis and Particle Size

The thermogravimetric analysis of coal fly ash was performed on air atmosphere, with a 10°C/min heating ratio, from 35°C to 1200°C, using Shimadzu DTG 60 equipment. The granulometric distribution was obtained by a CILAS 920 laser particles analyzer using ethanol as a dispersing agent.

#### 2.3.3. Surface Area

In order to perform analyses of surface area, ceramic samples were dried in vacuum at 100°C for 24 h. Nitrogen isotherms at 77 K were obtained in a Quantachrome Autosorb-1-MP in the partial pressure (P/P0) range 10–6–1. The specific surface area (SBET) was obtained using the Brunauer-Emmett-Teller (BET) method with 10 points [24].

### 2.4. Immobilization of Invertase

The immobilization of invertase in ceramic samples was performed as described by Albertini et al. [22]. Briefly, the ceramic (26.1 mg) was submerged in 2 mL of acetone with APTES (2.12 mM) at 80°C for 12 h. At the end of the reaction period, the sample was washed 5 times with ultrapure water and placed in 2 mL sodium phosphate buffer (10 mM, pH 7.0) with glutaraldehyde (1.92 mM) at 4°C for 12 h. Then, the sample was washed 5 times with buffer and dried at 60°C for 1 h, and the ceramic-activated surface was submerged in 1 mL of phosphate buffer containing invertase (5.40 mg) at 4°C for 4 h. After a covalent coupling period, the ceramic-invertase (ceramic with immobilized enzyme) was washed 5 times with NaCl (10 mM) and stored in ultrapure water at 4°C. The amount of immobilized enzyme on the surface of activated supports (mg of enzyme per g of support) was estimated by measurement of free protein concentration in the supernatant and washing solutions [21].

### 2.5. Hydrolysis of the Sucrose

#### 2.5.1. Activities of the Ceramic-Invertase and Free Enzyme

Assays of sucrose hydrolysis with ceramic-invertase samples and free enzyme were carried out as described by Albertini et al. [22]. Ceramic-invertase or free enzyme was incubated in a batch bioreactor containing 3.0 mL of sucrose solution (600 mg/mL) prepared in 100 mM sodium citrate buffer, pH 5.0, at 50°C under controlled agitation (150 rpm). The reducing sugars were then analyzed by the dinitrosalicylic acid (DNS) method [25] to obtain the percentage of sucrose hydrolyzed at intervals of 4, 8, 16, 32, and 64 minutes.

#### 2.5.2. Reusability of Ceramic-Invertase

The reuse stability of ceramic-invertase samples in a batch bioreactor was assayed under the same experimental condition that was employed in the activity assays. The ceramic-invertase was washed with ultrapure water after the first use and again incubated with sucrose. This procedure was repeated four times in a total of five hydrolysis assays to the same ceramic-invertase (four reuses).

#### 2.5.3. Storage of Ceramic-Invertase

After immobilization, ceramic-invertase samples were maintained in 100 mM sodium citrate buffer, pH 5.0, at 4°C for 30 days to perform assays on sucrose hydrolysis. Stability in the storage of ceramic-invertase samples was assayed under the same experimental conditions as previously stated.

#### 2.5.4. Contamination of Hydrolyzed Sucrose

The presence of residual invertase after hydrolysis experiments was estimated by measurement of free protein concentration in the hydrolyzed solution [21].

### 2.6. Statistical Analysis

Results of coal fly ash and ceramics characterization were obtained from five repetitions (*n* = 5). Data regarding the tests of immobilization, activity, reuse, and storage of invertase were obtained from ten repetitions (*n* = 10). All results were expressed as mean values. The Mann-Whitney nonparametric test was performed to determine significant differences between two mean values. The significance level used was 0.01 (*P* < 0.01). Statistical analyses were performed using OriginPro 8.5 software (OriginLab Corporation) and Statistica 10 software (StatSoft, Inc.).

## 3. Results and Discussion

### 3.1. Characterization of the Coal fly Ash

**Figure 1:**
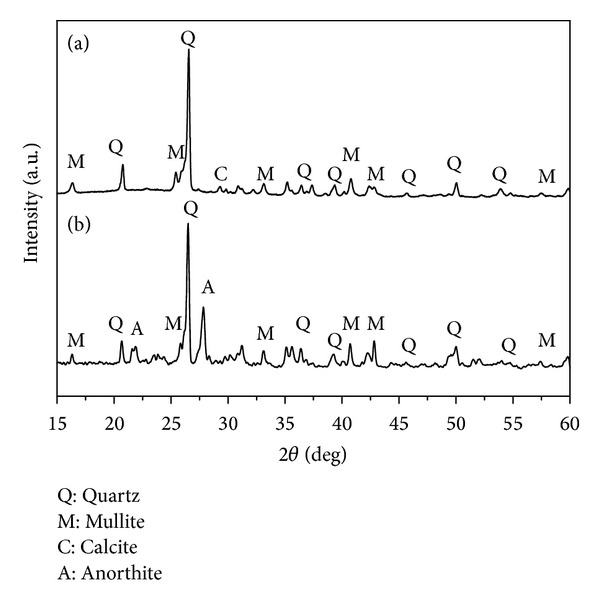
X-ray diffraction showing the main crystalline phases of (a) coal fly ash and (b) coal fly ash ceramic C318.

The XRD diffractogram of coal fly ash is shown in Figure 1(a). Crystallinity was around 62.9%, indicating the presence of amorphous phases (37.1%). The qualitative and quantitative crystallographic analyses resulted in mullite (36.8%), quartz (23.9%), and calcite (1.3%), as the main crystalline phases. Anhydrite, sillimanite, corundum, hematite, and anorthite were also identified in small concentrations (less than 1%). The degree of adjustment (*χ* ^2^) for structure refinement was 1.80 with residues RWP = 5.52 and REXP = 4.22. These results are similar to those obtained by Pires and Querol [26]. Coal fly ash is generally composed of mullite and quartz in a glassy matrix of aluminosilicates. Ash produced by thermal power plants in southern Brazil generally present a high content of SiO_2_ and a low amount of trace metals when compared with fly ash from other countries [7]. This feature is important in several applications [27].

**Figure 2:**
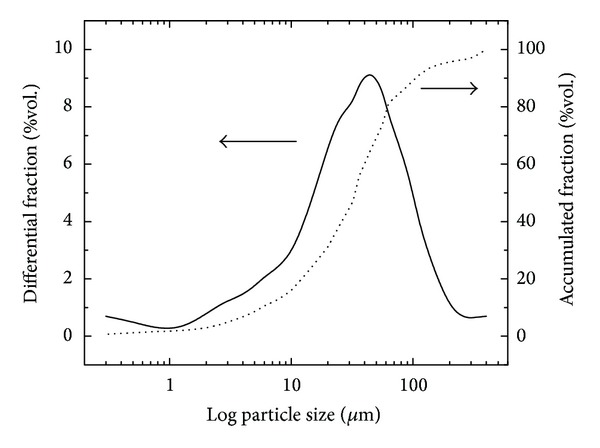
Particle size distribution and accumulated fraction of coal fly ash.

The particle size analysis (Figure 2) showed an approximate Gaussian asymmetric monomodal distribution, with particles having a diameter of 43.98 *μ*m. In the accumulated fraction, 10% of the particles had a size less than 6.00 *μ*m, 50% less than 68.26 *μ*m, and 90% less than 106.73 *μ*m. This distribution is typical of coal fly ash [26]. Particle size distribution plays an important role in the reactivity of the materials in transformation processes [28].

**Figure 3:**
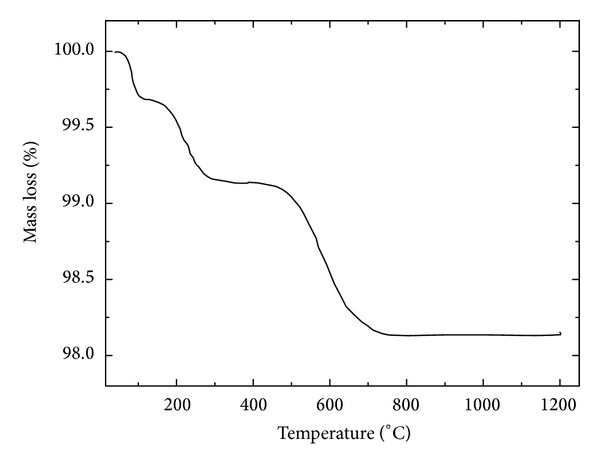
Thermogravimetric analysis of coal fly ash.

The thermogravimetric (TGA) curve (Figure 3) indicated a mass loss of 0.31% between room temperature and 100°C, which corresponds to loss of humidity; loss of hydration (0.55%) between 100 and 340°C; and loss of mass (1.12%) caused by the decomposition of CaCO_3_ and the burning of residual coal of the coal fly ash between 430 and 750°C. Above 750°C, the ash had thermogravimetric stability. Its small weight loss is associated with high ash content found in coal fly ash produced in southern Brazil [7]. Thermal events that occur in the coal fly ash are important in applications involving heat treatment.

### 3.2. Preparation and Characterization of Ceramics

**Figure 4:**
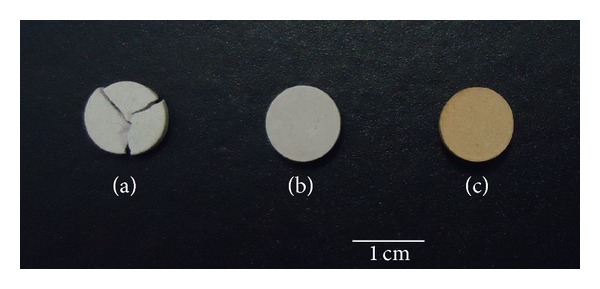
Preparation of coal fly ash ceramics: (a) fracture in pellet compacted at 198.98 MPa; (b) pellet compacted at 318.30 MPa; and (c) ceramic prepared with pellet compacted at 318.30 MPa.

After compaction, pellets prepared at pressures below 254.64 MPa (Figure 4(a)) presented fractures when removed from the steel mold. The low humidity of coal fly ash should have favored the poor adhesion of the particles under these pressures. Pellets at pressures above 190.98 MPa (Figure 4(b)) had the mechanical strength required for extraction from the steel mold. Thus, only green bodies compacted at 254.64 (C250) and 318.30 (C318) MPa were sintered at 1200°C for 4 h. After thermal treatment, ceramic pellets (Figure 4(c)) presented diameter, thickness, and mass of approximately 10 mm, 1 mm, 26.1 mg, respectively.

The XRD diffractogram of ceramic C318 is shown in Figure 1(b). This ceramic did not show statistically significant difference in crystallinity (around 63.1%) when compared to the coal fly ash. The thermal decomposition of calcite and residual coal (present in low concentrations) did not change the crystallinity of the pellet after sintering. The qualitative and quantitative crystallographic analyses resulted in mullite (36.5%), quartz (23.4%), and anhydrite (1.2%), as main crystalline phases. The degree of adjustment (*χ* ^2^) for structure refinement was 2.1 with residues RWP = 6.01 and REXP = 5.10. The concentrations of mullite and quartz in the ceramics did not show statistical difference (*P* > 0.01) when compared to the coal fly ash. This result was caused by the high thermal stability of these minerals. An increase in the concentration of anorthite was observed and was probably caused by diffusion of CaO from the decomposition of CaCO_3_, SiO_2_, and Al_2_O_3_ in grain boundaries during sintering. Similar results were obtained for ceramic C254.

A statistically significant increase was noted in the surface area when the compaction pressure was increased. SBET values to C254 and C318 were 16 m^2^/g and 35 m^2^/g, respectively. The increase in compaction pressure may have contributed to the formation of new surfaces from the fragmentation of grains [29]. This could explain the increase in surface area.

### 3.3. Immobilization of Invertase

It was seen that the amount of immobilized enzyme was significantly increased with increasing compaction pressure. Ceramics C254 and C318 immobilized 0.91 and 2.00 mg of protein, respectively. These values correspond to approximately 34.8 and 76.6 mg of enzyme per g of substrate. Supports that showed good results in immobilization processes as nylon-6 microbeads [30], magnetic PVAL microspheres [31], and montmorillonite [32] immobilized, respectively, at 4.95, 7.18, and 10 mg of enzyme per g of substrate. Thus, by comparison, the immobilization results presented by ceramics C254 and C318 are very promising. The presence of a CaO-Al_2_O_3_-SiO_2_ system in coal fly ash ceramics favors silanization, that is, self-assembled surface covering using organofunctional alkoxysilane, or, in this case, the covalent binding of the alkyl amine to glutaraldehyde and subsequent attachment of the enzyme to glutaraldehyde via Schiff's base linkage [33]. Ceramic C318 with the larger surface area had increased amounts of immobilized enzyme, a characteristic which plays an important role in the activation of the support and subsequent immobilization. In fact, the surface area was previously found to have a direct effect on the immobilization of invertase onto a substrate [34]. In the present work, it is important to observe that the ceramics maintained their physical integrity without fracture or fragmentation during the immobilization process, which is a consequence of good mechanical resistance after sintering. Based on the immobilization results, hydrolysis assays were performed only with C318-invertase having the larger amount of immobilized enzyme.

### 3.4. Hydrolysis of Sucrose

**Figure 5:**
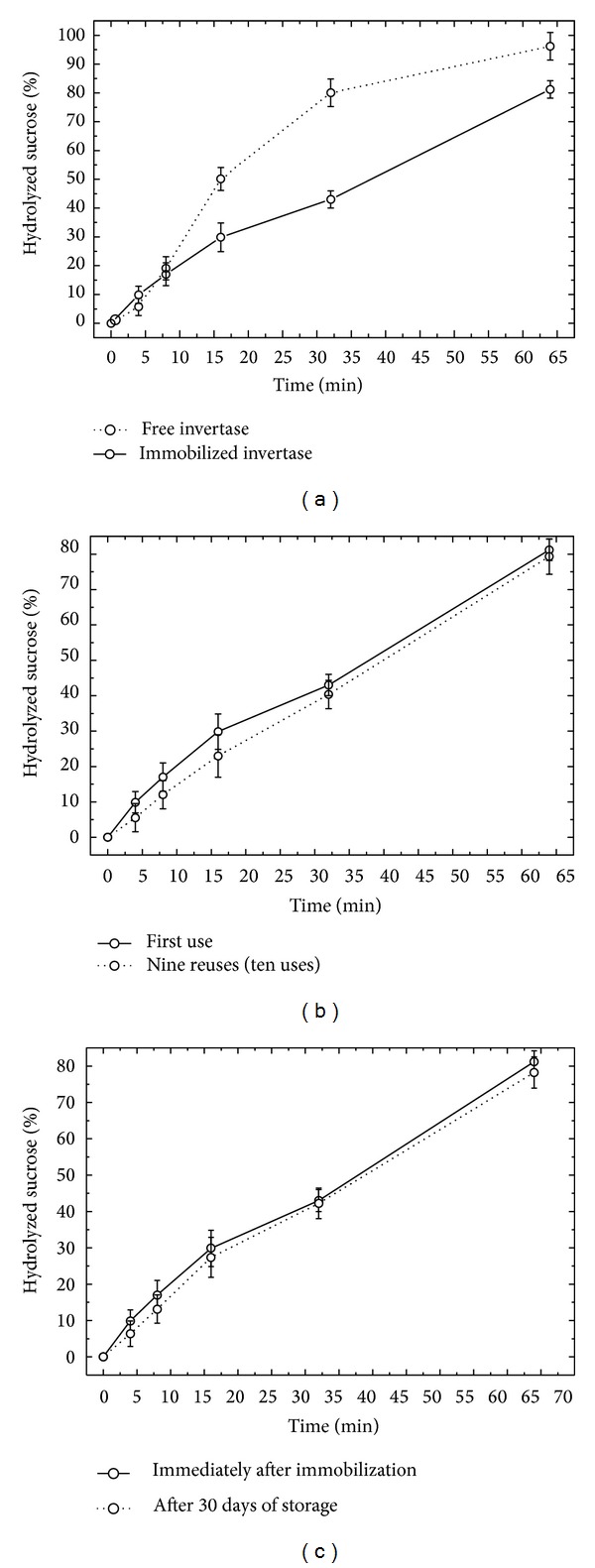
Results of assays of hydrolysis of the sucrose with the C318-invertase, after 65 min. (a) Comparative effect between free and immobilized invertase on the percentage of hydrolysed sucrose (mean ± standard deviation). (b) Effect of 9 reuses (10 uses) on the percentage of hydrolyzed sucrose (mean ± standard deviation). (c) Effect of storage (30 days) on the percentage of hydrolyzed sucrose (mean ± standard deviation).

The results of sucrose hydrolysis with free enzyme and C318-invertase after 65 min are shown in Figure 5(a). Two mg of free enzyme (mean amount of immobilized enzyme in C318-invertase) was used for comparison. After 65 min, the free enzyme and C318-invertase hydrolyzed, respectively, 96.2 and 81.2% of the sucrose. Although the differences in performance were statistically significant (15% less when compared with free enzyme), the immobilized enzyme still retained a high degree of hydrolysis. This performance remained approximately constant, even after nine reuses (ten experiments of hydrolysis with the same ceramic), as shown in Figure 5(b). C318-invertase ceramics showed a percentage of hydrolysis 14.6% lower and similar performance in reuse when compared to coal fly ashes glass-ceramic supports with zinc sulfate (GCSZn) [21, 22]. After 30 days' storage in buffer solution, C318-invertase showed enzymatic activity similar to that found immediately after the immobilization process (Figure 5(c)). Thus, the immobilized enzyme in ceramic C318 showed high activity and stability for reuse and storage. The presence of residual invertase was not detected in the reducing sugars. In addition, no fracture or fragmentation of the supports was noticed during the hydrolysis experiments. These results were similar to those of other investigators who also reported that supports are considered efficient for immobilization of invertase [21, 32].

**Figure 6:**
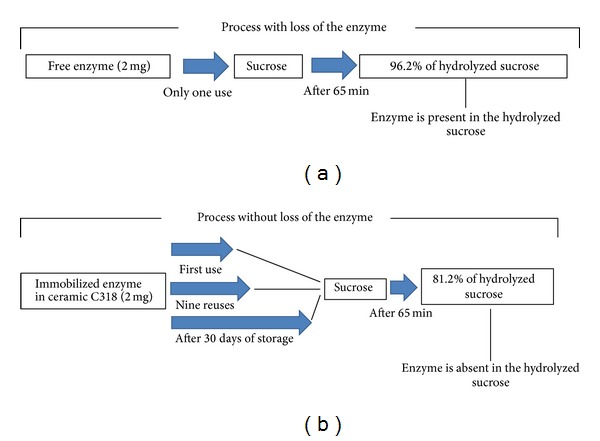
Schematic representation of the results from hydrolysis experiments: (a) free enzyme and (b) immobilized enzyme in C318.

A summary of results found in assays of hydrolysis is shown in Figure 6. The use of immobilized enzyme on C318, as compared with the free enzyme, demonstrates obvious advantages. Nevertheless, the next step in the use of this ceramic is the development of industrial bioreactors to provide efficient operation and significant increase of productivity.

## 4. Conclusions

Coal fly ash from the President Medici Thermal Power Plant was successfully used for the preparation of ceramic supports for the immobilization of invertase and hydrolysis of sucrose. The best support was C318 (compacted at 318.30 MPa), which showed a larger surface area and amount of immobilized enzyme. C318-invertase presented a high degree of hydrolysis when compared with the same amount of free enzyme. C318-invertase was shown to have high stability for reuse and storage. This can be a valuable alternative for the production of inverted sugar on an industrial scale through the use of bioreactors with C318-invertase.
